# Advanced glycation end products and RAGE signaling in diabetic cardiomyopathy: distinct mechanisms, human evidence, and translational barriers

**DOI:** 10.3389/fcvm.2026.1846184

**Published:** 2026-08-20

**Authors:** Zhiqiang Wang, Qingbo Shi, Xiaoya Li, Zhiwen Zhang, Muwei Li

**Affiliations:** 1 Health Science Center, Yangtze University, Jingzhou, China; 2 Department of Cardiology, Fuwai Central China Cardiovascular Hospital, Central China Fuwai Hospital of Zhengzhou University, Zhengzhou, China

**Keywords:** advanced glycation end products, biomarker stratification, diabetic cardiomyopathy, dicarbonyl stress, glyoxalase-1, myocardial stiffness, RAGE-dependent signaling, receptor for advanced glycation end products

## Abstract

Diabetic cardiomyopathy refers to myocardial structural, functional, and metabolic abnormalities enriched in people with diabetes that are not fully explained by obstructive epicardial coronary artery disease, significant valvular disease, or other established cardiac conditions. In clinical practice, diabetic myocardial dysfunction frequently overlaps with obesity, hypertension, chronic kidney disease, coronary microvascular dysfunction, and heart failure with preserved ejection fraction (HFpEF)-type cardiometabolic phenotypes. Among the mechanisms implicated in this condition, advanced glycation end products (AGEs) and receptor for advanced glycation end products (RAGE) signaling constitute a biologically plausible framework linking chronic hyperglycemia and dicarbonyl stress to myocardial injury. This review distinguishes RAGE-independent AGE-mediated injury, including extracellular matrix cross-linking and intracellular protein modification, from RAGE-dependent multiligand signaling, which may amplify oxidative, inflammatory, mitochondrial, calcium-handling, endothelial, and profibrotic responses. We further discuss RAGE as a multiligand pattern-recognition receptor and its interactions with danger signaling, innate immune pathways, and inflammasome activation within a broader inflammatory–oxidative network. In addition, we evaluate therapeutic strategies aimed at reducing glycation burden, interrupting matrix cross-linking, or inhibiting receptor-related signaling, and assess circulating and tissue-related biomarkers for phenotypic stratification and therapeutic monitoring. The review further considers methylglyoxal, glyoxal, 3-deoxyglucosone, and glyoxalase-1-dependent detoxification as components of dicarbonyl stress. Although experimental evidence supports biological plausibility, human evidence remains predominantly associative, circulating biomarkers lack cardiac specificity, and DCM-specific therapeutic trials are scarce. Future studies should use phenotype-enriched, multimodal biomarker strategies and mechanism-matched endpoints to determine whether selected patient subgroups may benefit from AGE- or RAGE-directed intervention.

## Introduction

Diabetic cardiomyopathy (DCM) is increasingly used to describe myocardial structural, functional, and metabolic abnormalities in people with diabetes that are not fully explained by obstructive epicardial coronary artery disease, significant valvular disease, or other established cardiac conditions (1–5). In clinical practice, however, DCM commonly overlaps with hypertension, obesity, chronic kidney disease, coronary microvascular dysfunction, and heart failure with preserved ejection fraction (HFpEF)-type cardiometabolic features, which complicates pathway-specific attribution and contributes to phenotypic heterogeneity (3–5). Clinically, DCM often evolves insidiously, with early abnormalities in relaxation, myocardial stiffness, and diastolic performance preceding overt systolic deterioration and symptomatic heart failure (2–6). This prolonged subclinical phase is highly relevant because it contributes to delayed recognition and may allow progressive myocardial injury to accumulate before conventional clinical markers become clearly abnormal (3–6). At the mechanistic level, DCM is now understood as a multifactorial process involving metabolic inflexibility, oxidative stress, mitochondrial dysfunction, inflammation, impaired calcium handling, and extracellular matrix remodeling rather than a single isolated defect (2–12).

Among the mechanisms implicated in this process, advanced glycation end products (AGEs) and receptor for advanced glycation end products (RAGE) signaling constitute biologically plausible, interacting contributors linking chronic hyperglycemia and dicarbonyl stress to myocardial injury (13–18). Their effects should be separated into RAGE-independent structural and intracellular protein modifications and RAGE-dependent receptor signaling; these processes are complementary but not interchangeable (14–20). AGE-related injury should therefore be regarded as a potential contributor to, rather than a universal driver of, cardiac remodeling and functional deterioration (13–20).

RAGE is a multiligand pattern-recognition receptor that may amplify receptor-dependent inflammatory and oxidative responses in the diabetic myocardium (14–17, 21, 22). Human studies primarily support associations between tissue AGE burden, diastolic dysfunction, and reduced exercise capacity, whereas evidence that interrupting AGE formation, matrix cross-linking, or RAGE-related signaling improves myocardial outcomes is predominantly preclinical (14, 15, 19–24).

Despite this mechanistic rationale, the translational trajectory of the AGE–RAGE axis in DCM remains incomplete (3, 4, 10, 15, 24). Current clinical management still relies primarily on glycemic optimization and standard cardiometabolic therapy rather than mechanism-based anti-glycation strategies, and most candidate interventions directed at AGE accumulation, matrix cross-linking, or receptor signaling remain limited to preclinical or early translational stages (10, 15, 24). These limitations indicate that the central questions are how much pathway-specific causal weight AGE-mediated and RAGE-dependent mechanisms carry in human DCM, which phenotypes are most strongly enriched for these mechanisms, and how target engagement can be assessed in clinical studies. Accordingly, this review critically evaluates their mechanistic plausibility, the hierarchy and limitations of human evidence, biomarker specificity, and requirements for phenotype-guided trial design (14, 15, 21–24).

## AGE formation, accumulation, and homeostatic imbalance in diabetes

### Non-enzymatic glycation, reactive dicarbonyl stress, and glyoxalase-dependent detoxification

Non-enzymatic glycation is the core biochemical process underlying AGE formation and represents a direct molecular link between chronic hyperglycemia and tissue injury in diabetes (25–29). In the initial stage of this reaction, reducing sugars react non-enzymatically with free amino groups on proteins, lipids, and nucleic acids to form reversible Schiff bases, which subsequently rearrange into more stable Amadori products (25–28). Under sustained metabolic stress, these early glycation intermediates undergo oxidation, dehydration, and fragmentation, generating a heterogeneous group of largely irreversible AGE adducts with distinct structural and biological properties (25–29). This process is not merely a passive chemical consequence of elevated glucose exposure; rather, it progressively alters protein charge, conformation, turnover, and intermolecular interactions, thereby providing a biochemical basis for persistent myocardial vulnerability in diabetes (26–29).

In addition to glucose itself, reactive dicarbonyls—including methylglyoxal (MGO), glyoxal, and 3-deoxyglucosone (3-DG)—are major accelerators of glycation (25–31). MGO arises largely from glycolytic intermediates, glyoxal can be generated through carbohydrate oxidation and lipid peroxidation, and 3-DG is produced during degradation of Amadori intermediates (25–31). Because these species are substantially more reactive than glucose, they can directly modify intracellular proteins before or independently of stable extracellular AGE cross-link formation, linking dicarbonyl stress to protein dysfunction as well as to subsequent AGE accumulation (27, 30, 31). In the diabetic myocardium, this biochemical environment is especially relevant because it increases the burden of structurally damaging and signaling-competent AGE species, thereby creating the upstream conditions for subsequent matrix stiffening, receptor engagement, and maladaptive remodeling (27–31).

Glyoxalase 1 (GLO1) is the rate-limiting enzyme of the glutathione-dependent glyoxalase system and is a major determinant of cellular MGO detoxification (30, 31). Reduced GLO1 capacity or an imbalance between dicarbonyl generation and detoxification may expand the dicarbonyl proteome, enhance intracellular protein modification, and increase the substrate available for AGE formation (30, 31). Experimental GLO1 overexpression attenuates oxidative injury in diabetic myocardium, but direct evidence linking myocardial GLO1 activity to DCM phenotypes or clinical outcomes in humans remains limited (31).

### Myocardial AGE accumulation and structural consequences

In the diabetic heart, AGE-related injury should be separated into RAGE-independent structural effects and RAGE-dependent signaling effects (14, 15, 19, 20, 23). The non-enzymatic cross-linking of collagen and other long-lived matrix proteins is primarily a receptor-independent chemical process that increases extracellular matrix stiffness, reduces ventricular compliance, and impairs diastolic relaxation (19, 20, 29). Because these collagen cross-links are chemically stable and poorly reversible, AGE accumulation creates a durable structural substrate for myocardial fibrosis and stiffness, thereby helping to explain why diastolic dysfunction may progress even when glycemic control improves later in the disease course (14, 15, 19, 29).

Separately, selected AGE species can engage RAGE and activate receptor-dependent inflammatory, oxidative, and profibrotic signaling (14, 15, 32). In parallel, intracellular glycation and dicarbonyl-derived adducts may impair calcium-regulatory proteins, including sarcoplasmic/endoplasmic reticulum calcium ATPase 2a (SERCA2a) and ryanodine receptor 2 (RyR2); these effects should not be assumed to require RAGE unless receptor dependence has been directly demonstrated (23, 33–35).

Although AGEs comprise a chemically heterogeneous group of adducts, only a subset appears particularly relevant to DCM (14, 15, 18–20, 23). Among the non-cross-linking species, N*ε*-carboxymethyl-lysine and N*ε*-carboxyethyl-lysine are informative markers of chronic glycoxidative or dicarbonyl burden and remain biologically relevant because they modify protein structure and contribute to ligand recognition (14, 15, 18–20). By contrast, methylglyoxal-derived hydroimidazolones are more closely linked to active intracellular carbonyl stress and calcium-handling abnormalities, whereas pentosidine-like cross-linking AGEs are especially important because they directly increase collagen cross-linking, extracellular matrix stiffness, and ventricular rigidity (18–20, 23, 33–35). The major AGE species most relevant to DCM and their principal myocardial implications are summarized in Table 1 (14, 15, 18–20, 23, 33–35).

**Table 1:** Major AGE species relevant to diabetic cardiomyopathy and their RAGE-independent structural or signaling-related implications.

AGE species	Main precursors/formation pathway	Key structural/biological features	Principal relevance to diabetic cardiomyopathy	Ref.
N*ε*-carboxymethyl-lysine	Generated from glucose-derived Amadori products through glycoxidation and lipid peroxidation; abundant in tissues and circulation	Non-cross-linking, non-fluorescent AGE associated with protein modification and receptor for advanced glycation end products-related inflammatory signaling	Reflects chronic glycoxidative stress and is associated with myocardial stiffness, diastolic dysfunction, and adverse remodeling	(14, 15, 19, 20)
Nε-carboxyethyl-lysine	Formed mainly by reaction of methylglyoxal with lysine residues; marker of dicarbonyl stress	Non-cross-linking AGE that accumulates in intracellular and extracellular proteins and alters protein function	Indicates dicarbonyl-mediated myocardial injury and is associated with fibrosis and impaired diastolic function	(14, 15, 18, 23)
Methylglyoxal-derived hydroimidazolones	Formed when methylglyoxal reacts with arginine residues under hyperglycemic and oxidative conditions	Highly reactive dicarbonyl-derived adducts linked to intracellular protein dysfunction and oxidative stress	Associated with impaired calcium handling, reduced contractile reserve, and early excitation–contraction abnormalities	(18, 23, 33–35)
Cross-linking advanced glycation end products (for example, pentosidine)	Generated through glycoxidation-related reactions and preferentially accumulate in long-lived extracellular matrix proteins such as collagen	Fluorescent, strongly cross-linking AGE species that increase matrix stiffness and reduce tissue elasticity	Provide a structural basis for myocardial stiffening, reduced ventricular compliance, and diastolic dysfunction	(19, 20, 29)

AGE, advanced glycation end product; RAGE, receptor for advanced glycation end products; MGO, methylglyoxal; 3-DG, 3-deoxyglucosone; GLO1, glyoxalase 1.

Cross-linking AGEs primarily exert RAGE-independent structural effects on long-lived extracellular matrix proteins, whereas non-cross-linking AGE species may indicate glycoxidative or dicarbonyl burden and may also participate in receptor-dependent signaling. MGO-derived hydroimidazolones represent active dicarbonyl-related protein modification; glyoxal, 3-DG, and GLO1 are discussed in the main text because they describe upstream dicarbonyl generation or detoxification rather than individual AGE species.

Preclinical studies further suggest that the structural component of AGE-mediated injury may be at least partially modifiable (36–38). AGE cross-link breakers such as alagebrium have been shown to reduce collagen cross-linking, improve ventricular compliance, and attenuate diabetes-related cardiac dysfunction in experimental models (36–38). Taken together, current evidence supports two distinct but interacting routes: receptor-independent matrix cross-linking and intracellular protein modification, and receptor-dependent inflammatory and oxidative signaling (14, 15, 19, 20, 32, 36–38). This model provides a biologically plausible framework, but the relative contribution of each route to human DCM remains incompletely defined.

### AGE clearance and homeostatic imbalance in diabetes

The biological burden of AGEs is determined not only by their rate of formation but also by the efficiency of their systemic clearance (39–42). Under physiological conditions, renal excretion is a major route for the elimination of circulating AGE adducts and AGE-modified peptides, thereby limiting their residence time in the circulation and reducing tissue deposition (39–41). In addition, cellular uptake and intracellular degradation, including pathways related to advanced glycation end product receptor 1, may further restrain AGE-related oxidative and inflammatory stress (39, 42). From a homeostatic perspective, these mechanisms help maintain a dynamic balance between continuous AGE generation and removal (39–42).

In diabetes, this balance becomes progressively disrupted (18, 39–41). Chronic hyperglycemia and dicarbonyl stress increase AGE formation, while aging and declining renal function reduce clearance capacity, together favoring net AGE retention (18, 39–41). As circulating AGEs accumulate, tissue deposition and ligand availability may increase; however, systemic AGE retention should not be interpreted as a cardiac-specific measure of myocardial RAGE activation, because renal function, age, vascular disease, and multiorgan glycation burden influence circulating concentrations (14, 15, 39–41).

**Figure 1:**
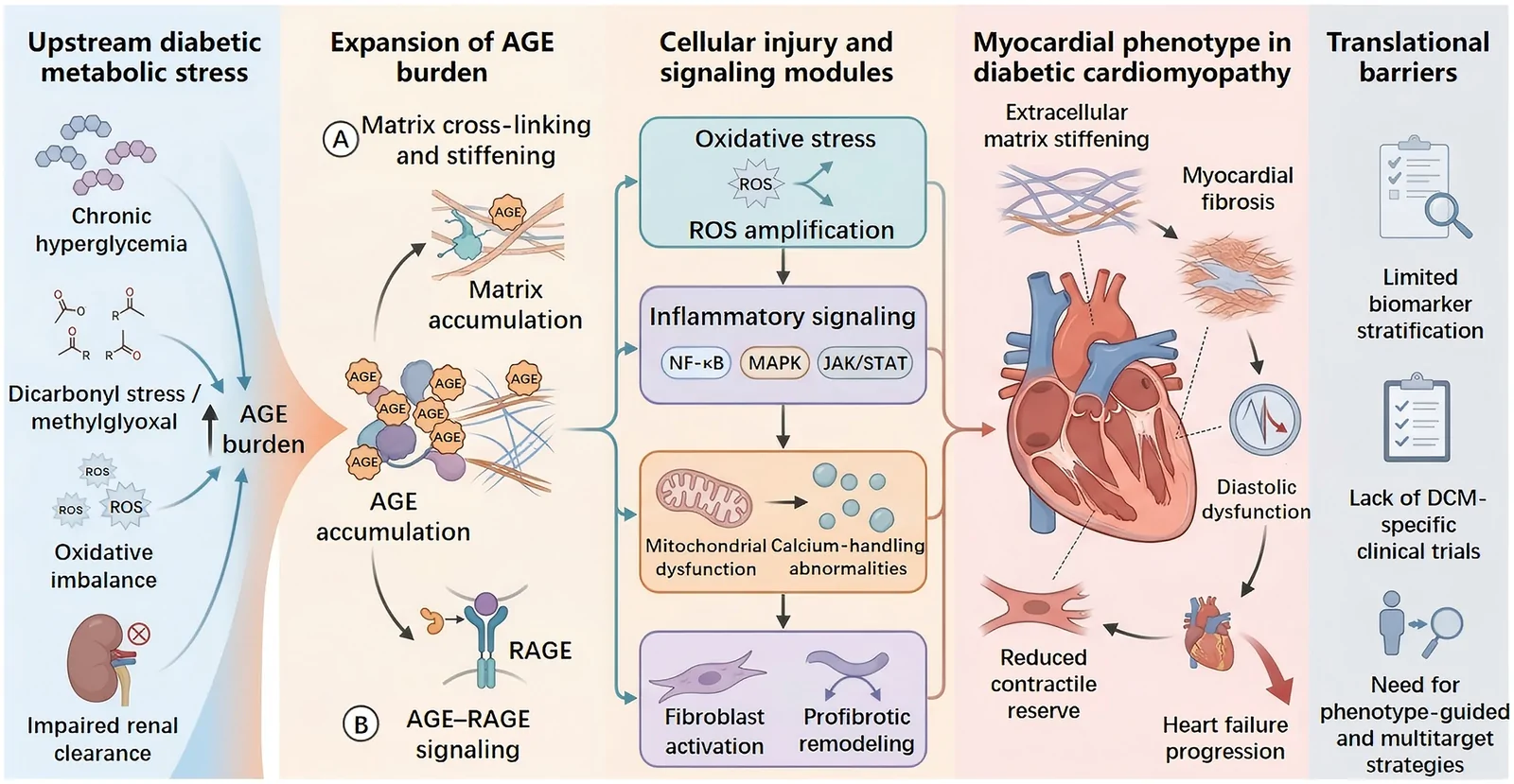
Integrated framework linking diabetic metabolic and dicarbonyl stress to AGE-mediated myocardial remodeling and translational barriers. AGE, advanced glycation end product; RAGE, receptor for advanced glycation end products; DCM, diabetic cardiomyopathy; ROS, reactive oxygen species; NF-*κ*B, nuclear factor kappa B; MAPK, mitogen-activated protein kinase; JAK/STAT, Janus kinase/signal transducer and activator of transcription; MGO, methylglyoxal; 3-DG, 3-deoxyglucosone; GLO1, glyoxalase 1. Diabetic metabolic stress expands AGE burden through chronic hyperglycemia, reactive dicarbonyl formation, oxidative imbalance, and impaired renal clearance. MGO is shown as a representative dicarbonyl; the broader category also includes glyoxal and 3-DG, while impaired GLO1-dependent detoxification may further increase dicarbonyl burden. The schematic distinguishes RAGE-independent matrix cross-linking/stiffening from RAGE-dependent ligand signaling. These mechanisms may contribute to myocardial remodeling and functional deterioration, but their pathway-specific causal contribution in human DCM remains incompletely established.

This homeostatic imbalance is particularly relevant in diabetic cardiorenal disease, where enhanced AGE formation and impaired clearance may interact to accelerate fibrosis, stiffness, and functional deterioration (24, 39–41). Accordingly, strategies aimed at lowering systemic AGE burden should target not only glycation chemistry itself, but also dicarbonyl detoxification, renal preservation, and endogenous clearance pathways (18, 24, 39–42). The integrated progression from diabetic metabolic stress to AGE burden expansion and downstream myocardial consequences is summarized in Figure 1 (14, 15, 18, 24, 39–42).

## RAGE biology in the diabetic heart

### Structure and expression of RAGE

RAGE is a transmembrane pattern-recognition receptor that provides a structural platform through which AGEs and other ligands may initiate and sustain receptor-dependent cellular stress responses in DCM (21, 43, 44). Its extracellular region consists of one variable (V) domain and two constant domains (C1 and C2), which together form the principal ligand-binding interface (43, 44). Within the V domain, conserved residues contribute to protein folding, structural stability, and ligand recognition, whereas the short cytoplasmic tail is essential for intracellular signal propagation (44, 45). This structural organization supports the concept that RAGE can function as a signaling receptor capable of sustaining maladaptive responses when ligand burden increases (21, 44, 45).

In the heart, RAGE is expressed in several cell types relevant to DCM, including cardiomyocytes, fibroblasts, endothelial cells, and inflammatory cells (21, 46). Under diabetic and inflammatory conditions, both ligand availability and receptor expression increase, thereby sensitizing the myocardium to sustained downstream activation (21, 46, 47). In addition, post-translational modification of the extracellular region, particularly glycosylation, may influence ligand affinity and signaling strength, suggesting that diabetic stress affects not only receptor abundance but also receptor behavior (48). Following ligand engagement, the RAGE cytoplasmic tail interacts with diaphanous-related formin 1 (DIAPH1), an intracellular effector that links receptor activation to Rac1/Cdc42-related cytoskeletal and migratory signaling (45, 49–52). Because this interaction is repeatedly invoked in the mechanistic and therapeutic sections, its molecular role and evidence limitations are described separately below.

### RAGE–DIAPH1 intracellular signaling interface

DIAPH1 is not a RAGE ligand; rather, it binds the short cytoplasmic tail of RAGE and helps couple extracellular ligand recognition to intracellular signaling (45, 49–52). This interface can engage Rac1 and Cdc42, influence cytoskeletal organization and cell migration, and support downstream oxidative and inflammatory responses (45, 49–52). Disrupting RAGE–DIAPH1 interaction is therefore mechanistically distinct from blocking extracellular ligand binding. However, the supporting evidence is predominantly cellular and animal-based, and direct demonstration of myocardial RAGE–DIAPH1 target engagement or pathway-specific causality in patients with DCM is currently lacking (49–52).

**Figure 2:**
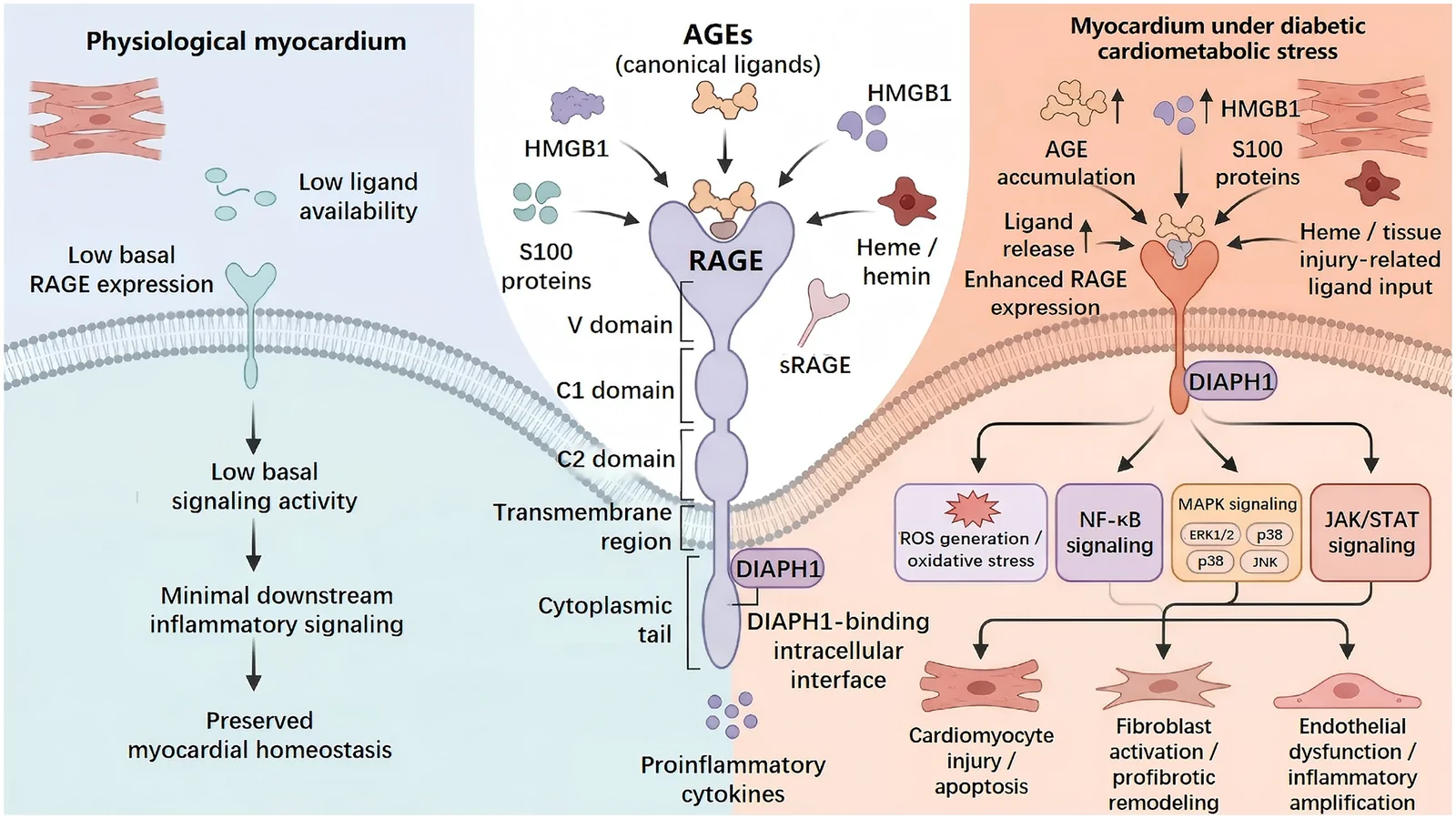
Structural organization of RAGE and multiligand signaling under physiological conditions and diabetic cardiometabolic stress. AGE, advanced glycation end product; RAGE, receptor for advanced glycation end products; sRAGE, soluble receptor for advanced glycation end products; HMGB1, high-mobility group box 1; DIAPH1, diaphanous-related formin 1; ROS, reactive oxygen species; NF-*κ*B, nuclear factor kappa B; MAPK, mitogen-activated protein kinase; ERK1/2, extracellular signal-regulated kinase 1/2; JNK, c-Jun N-terminal kinase; JAK/STAT, Janus kinase/signal transducer and activator of transcription. The right panel depicts the broader diabetic cardiometabolic environment, which may include hyperglycemia, obesity, hypertension, renal dysfunction, lipotoxicity, and systemic inflammation. Increased AGEs and non-AGE ligands may enhance RAGE engagement and DIAPH1-dependent signaling. DIAPH1 is an intracellular signaling effector rather than a ligand, and evidence for myocardial RAGE–DIAPH1 signaling in human DCM remains predominantly preclinical and indirect. sRAGE is shown as a potential extracellular decoy receptor.

These structural and regulatory features support the concept that RAGE can function as a multiligand, context-dependent signaling receptor rather than solely as an AGE-binding molecule (21, 45, 47). Figure 2 contrasts low basal signaling with enhanced ligand-dependent activation under diabetic cardiometabolic stress, encompassing hyperglycemia, obesity, lipotoxicity, renal dysfunction, and systemic inflammation (21, 43–48).

### Ligand repertoire of RAGE

RAGE should not be viewed solely as an AGE receptor; it may function as a multiligand pattern-recognition receptor that integrates several injury-associated inputs and contributes to sustained inflammatory and oxidative signaling (21, 47, 53, 54).

AGEs are canonical RAGE ligands and may contribute to receptor-dependent injury in selected DCM phenotypes (14, 15, 21). High-mobility group box 1 (HMGB1) and S100 proteins are protein damage-associated molecular patterns (DAMPs), whereas cell-free heme and hemin are non-protein injury-associated RAGE ligands generated during hemolysis or tissue damage (47, 53–59). Their circulating concentrations may reflect systemic inflammation, vascular injury, hemolysis, or multiorgan stress and therefore should be considered candidate systemic injury markers rather than validated cardiac-specific biomarkers. The multiligand repertoire supports a role for RAGE as a potential convergence receptor, not proof that it is the dominant upstream determinant of human DCM (21, 47, 53–59).

### Downstream signaling mechanisms of RAGE

A critical intracellular step is the interaction between the RAGE cytoplasmic tail and DIAPH1 (45, 49–52). This interaction is mechanistically downstream of ligand binding and is distinct from extracellular ligand neutralization. It can support Rac1/Cdc42-related signaling and downstream inflammatory or oxidative responses; however, its role in human DCM has not been directly established (45, 49–52).

Among the downstream pathways activated by RAGE, nuclear factor kappa B is one of the most important mediators of persistent inflammatory signaling (21, 22, 47). RAGE activation also engages mitogen-activated protein kinase pathways, including extracellular signal-regulated kinase 1/2, p38 mitogen-activated protein kinase, and c-Jun N-terminal kinase, thereby reinforcing inflammatory gene expression, cellular stress responses, and fibroblast activation (21, 22, 47, 60). In parallel, RAGE signaling promotes reactive oxygen species generation through redox-sensitive mechanisms, creating a self-reinforcing loop in which oxidative stress further amplifies nuclear factor kappa B- and mitogen-activated protein kinase-dependent signaling (21, 22, 60). Janus kinase/signal transducer and activator of transcription signaling may also contribute to this downstream network, particularly under sustained diabetic stress, where receptor activation intersects with broader inflammatory and remodeling programs in the myocardium (21, 22).

These pathways provide a plausible molecular basis through which RAGE engagement by AGE and non-AGE ligands may contribute to mitochondrial dysfunction, calcium-handling abnormalities, cardiomyocyte injury, and fibroblast activation (21, 22, 47, 49–60). RAGE is therefore described here as a multiligand receptor within a broader signaling network, while RAGE-independent AGE effects are considered separately. The major ligand modules and receptor-dependent pathways are summarized in Table 2 (21, 22, 47, 49–60).

**Table 2:** Major RAGE ligand modules and receptor-dependent signaling pathways relevant to diabetic cardiomyopathy.

RAGE-related module	Representative ligands/sources	Representative downstream pathways	Principal relevance to diabetic cardiomyopathy	Ref.
AGE ligand–RAGE signaling module	AGE-modified proteins and matrix components, including Nε-carboxymethyl-lysine, Nε-carboxyethyl-lysine, and methylglyoxal-derived adducts	Reactive oxygen species generation, nuclear factor kappa B, mitogen-activated protein kinase, Janus kinase/signal transducer and activator of transcription, diaphanous-related formin 1-dependent signaling	May amplify oxidative and inflammatory signaling, fibroblast activation, and myocardial remodeling; pathway-specific causal effects in human DCM remain unproven	(14, 15, 21, 22, 49–52, 60)
High-mobility group box 1–RAGE/Toll-like receptor 4 co-axis	High-mobility group box 1 released from injured myocardium, necrotic cells, and activated immune cells	RAGE and Toll-like receptor 4/myeloid differentiation primary response 88 signaling, nuclear factor kappa B activation, proinflammatory cytokine amplification	Intensifies sterile inflammation, links tissue injury to sustained immune activation, and contributes to fibrotic remodeling	(55, 56)
S100 family–RAGE module	S100 proteins released by activated leukocytes and stressed tissues, especially S100 calcium-binding protein A12	Mitogen-activated protein kinase, nuclear factor kappa B, Janus kinase/signal transducer and activator of transcription	Contributes to vascular inflammation, endothelial dysfunction, and profibrotic signaling in diabetic and heart failure settings	(53, 54, 57)
Heme–RAGE module	Cell-free heme and hemin generated during hemolysis, bleeding, or tissue injury	Receptor oligomerization, oxidative stress, nuclear factor kappa B-related inflammatory signaling	May aggravate microvascular inflammation and tissue vulnerability under combined metabolic and vascular stress	(58, 59)

AGE, advanced glycation end product; RAGE, receptor for advanced glycation end products; HMGB1, high-mobility group box 1; DAMP, damage-associated molecular pattern.

This table focuses on RAGE-dependent signaling and does not include RAGE-independent AGE-mediated matrix cross-linking. HMGB1 and S100 proteins are protein DAMPs, whereas heme and hemin represent non-protein injury-associated RAGE ligands. The clinical biomarker value of these circulating ligands remains unvalidated and non-cardiac-specific.

## Distinct AGE-mediated and RAGE-dependent mechanisms contributing to myocardial dysfunction

### Cardiomyocyte dysfunction and calcium-handling abnormalities

In DCM, intracellular glycation/dicarbonyl-derived protein modification and RAGE-dependent signaling may each contribute to cardiomyocyte dysfunction through partly distinct mechanisms (10, 23, 52, 60). Experimental AGE exposure impairs SERCA2a, RyR2, calcium transient amplitude, and diastolic calcium reuptake (23, 33–35); however, these findings should be described as AGE-related experimental effects unless RAGE dependence has been directly demonstrated. Their contribution to human DCM is biologically plausible but remains incompletely quantified.

### Oxidative stress and mitochondrial dysfunction

Oxidative stress is an important shared downstream process in diabetic myocardium (8, 9, 12, 14, 15). RAGE engagement may increase reactive oxygen species (ROS) through redox-sensitive pathways, while intracellular glycation and mitochondrial dysfunction can independently add to oxidant burden (8, 9, 21–23, 60). These interacting processes may create a feed-forward environment of redox and metabolic impairment; however, pathway-specific causality in human DCM has not been established.

### Fibroblast activation, extracellular matrix remodeling, and stiffness

Fibrotic remodeling may involve two separable AGE-related components (15, 19, 20, 29, 32). First, RAGE-dependent inflammatory and transforming growth factor-beta (TGF-*β*)-related signaling may promote fibroblast activation and matrix production (15, 32, 60). Second, AGE-mediated collagen cross-linking is a RAGE-independent chemical modification that directly increases matrix rigidity and reduces ventricular distensibility (19, 29, 36–38). These components may interact pathophysiologically but should not be presented as one receptor pathway.

### Endothelial and microvascular contributions

Endothelial injury may be an important contributor to coronary microvascular dysfunction in diabetes (15, 21, 61). RAGE-dependent activation by AGE and non-AGE ligands may promote oxidative stress and inflammation, while lipotoxicity, hypertension, renal dysfunction, and other receptor systems also influence endothelial phenotype (53, 57–59, 61–64). Accordingly, RAGE-related signaling should be positioned as one component of a broader cardiometabolic microvascular network, not as the sole driver of endothelial injury.

## RAGE-dependent multiligand signaling within the inflammatory–oxidative stress network

### Inflammatory signaling downstream of RAGE

The following section focuses specifically on receptor-dependent signaling initiated by AGEs and non-AGE ligands; it does not encompass RAGE-independent AGE-mediated matrix cross-linking. Inflammatory activation is an important downstream output of sustained RAGE engagement and may contribute to progressive myocardial injury within a broader network of metabolic, mitochondrial, microvascular, and immune abnormalities (21, 22, 45, 47, 49–52, 60, 65, 66).

The inflammatory consequences of RAGE activation are tightly interwoven with oxidative stress, forming a self-sustaining network rather than a simple linear cascade (8, 21, 22, 60, 66, 67). AGE–RAGE signaling enhances reactive oxygen species generation through redox-sensitive pathways, while reactive oxygen species further potentiate nuclear factor kappa B- and mitogen-activated protein kinase-dependent inflammatory outputs (8, 21, 22, 60, 67). This reciprocal amplification is particularly relevant in diabetic cardiomyopathy, where chronic metabolic stress, mitochondrial dysfunction, and impaired antioxidant defenses already predispose the myocardium to a state of heightened inflammatory susceptibility (8, 9, 12, 60, 65, 66). Thus, low-grade inflammation in the diabetic heart should not be regarded as a passive epiphenomenon of injury, but as an organized and persistent signaling state maintained by ligand burden, receptor activation, and redox imbalance (21, 22, 49–52, 60, 65, 66).

**Figure 3:**
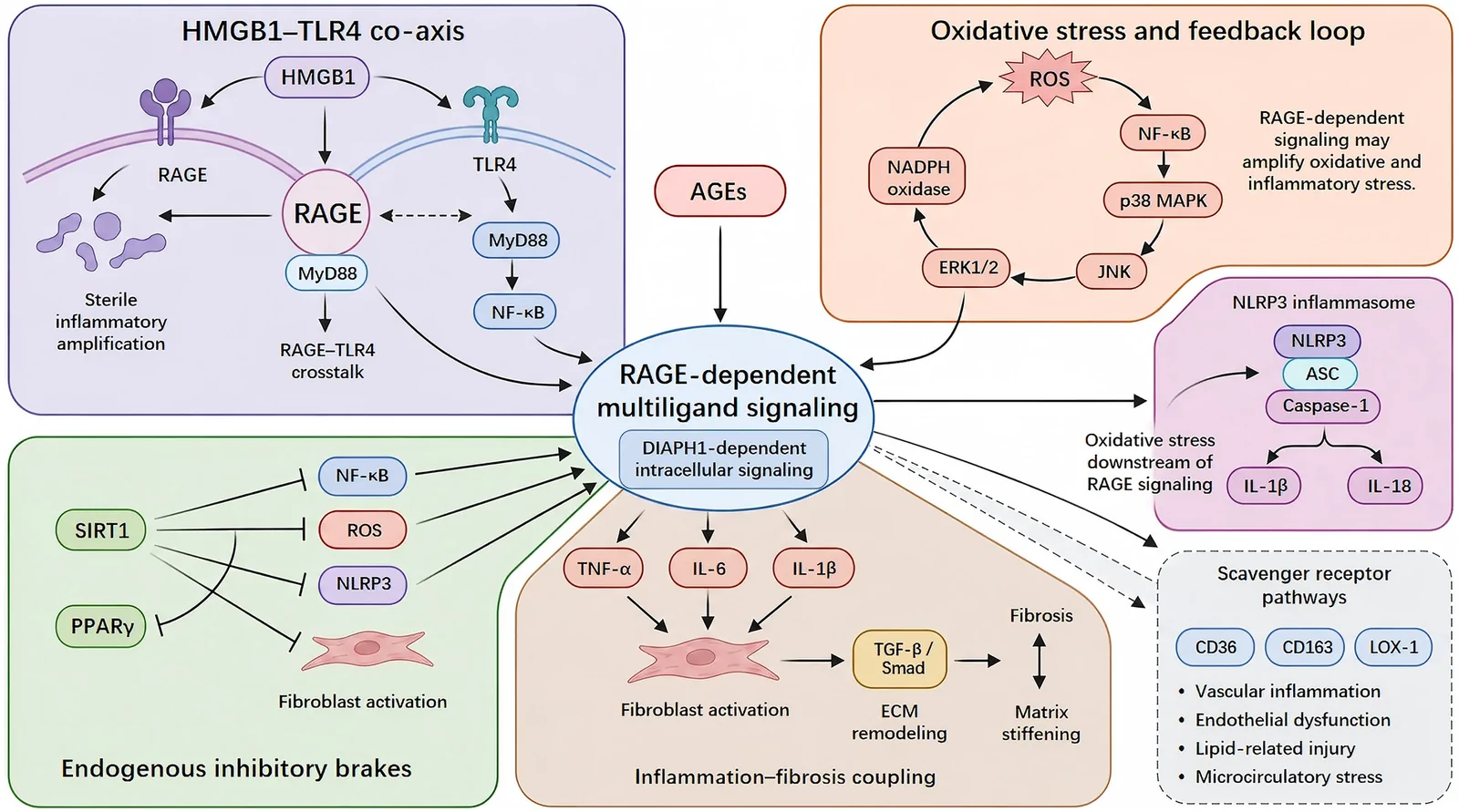
RAGE-dependent multiligand inflammatory–oxidative signaling network in diabetic cardiomyopathy. AGE, advanced glycation end product; RAGE, receptor for advanced glycation end products; TLR4, Toll-like receptor 4; MyD88, myeloid differentiation primary response 88; DIAPH1, diaphanous-related formin 1; ROS, reactive oxygen species; NF-*κ*B, nuclear factor kappa B; MAPK, mitogen-activated protein kinase; ERK1/2, extracellular signal-regulated kinase 1/2; JNK, c-Jun N-terminal kinase; NLRP3, NOD-like receptor family pyrin domain containing 3; ASC, apoptosis-associated speck-like protein containing a CARD; SIRT1, sirtuin 1; PPAR*γ*, peroxisome proliferator-activated receptor gamma; TNF-α, tumor necrosis factor-α; IL-6, interleukin-6; IL-1β, interleukin-1β; IL-18, interleukin-18; TGF-*β*, transforming growth factor-*β*; ECM, extracellular matrix; DCM, diabetic cardiomyopathy. This figure specifically summarizes receptor-dependent signaling initiated by AGEs and non-AGE DAMPs. RAGE-independent structural effects of AGEs, particularly extracellular matrix cross-linking and myocardial stiffening, are presented separately in Figure 1 and discussed in the main text. RAGE-dependent signaling interacts with HMGB1–TLR4 crosstalk, ROS–NF-*κ*B/MAPK feedback, and NLRP3 inflammasome activation and may contribute to inflammatory–fibrotic remodeling. The figure represents a mechanistic framework primarily supported by experimental evidence and does not establish RAGE as the dominant causal pathway in human DCM.

Importantly, inflammatory signaling downstream of RAGE cannot be interpreted solely in the context of AGE ligands themselves (47, 53–56, 67). High-mobility group box 1 can signal through both RAGE and Toll-like receptor 4, thereby intensifying sterile inflammation and extending the duration of inflammatory activation in injured myocardium (53–56). Moreover, recent evidence indicates that glycation-derived inflammatory signaling may also intersect with myeloid differentiation factor 2-dependent Toll-like receptor 4 activation, suggesting that AGE-driven myocardial inflammation is mediated through a broader innate immune signaling architecture than classical receptor for advanced glycation end products biology alone would imply (67). This receptor crosstalk supports viewing RAGE-dependent signaling as a potential convergence node within a broader innate immune and oxidative network, rather than as a universally dominant inflammatory hub (21, 22, 47, 53–56, 66, 67). The relative importance of RAGE, TLR4, inflammasome pathways, and parallel metabolic injury is likely to vary across DCM phenotypes. This receptor-dependent framework is illustrated in Figure 3 (21, 22, 49–56, 60).

### Coupling between inflammation and fibrosis

In DCM, inflammation and fibrosis are not sequentially related but biologically co-constructed processes that evolve through sustained reciprocity between cytokine signaling, fibroblast activation, and matrix remodeling (32, 60, 68–71). Proinflammatory mediators induced downstream of AGE–RAGE signaling, particularly tumor necrosis factor-alpha, interleukin-6, and interleukin-1 beta, do not merely mark immune activation; they actively reprogram cardiac fibroblasts toward a profibrotic phenotype characterized by enhanced proliferation, myofibroblast differentiation, and extracellular matrix turnover (32, 68–71). As a result, the diabetic myocardium gradually shifts from adaptive repair toward a state of persistent fibroinflammatory remodeling in which inflammatory signaling and matrix production become mutually reinforcing (32, 60, 68–70).

A pivotal bridge within this transition is the convergence of reactive oxygen species, inflammasome signaling, and transforming growth factor-beta-dependent profibrotic programming (60, 68, 72, 73). RAGE-related oxidative stress may create a permissive environment for NOD-like receptor family pyrin domain containing 3 inflammasome activation, whereas interleukin-1 beta release further amplifies fibroblast activation and matrix deposition (60, 68). In parallel, transforming growth factor-beta/Smad signaling functions as a canonical fibrotic effector pathway that converts inflammatory stress into sustained extracellular matrix accumulation and tissue stiffening (72, 73). This convergence is pathophysiologically important because it explains why chronic inflammatory activation in DCM often culminates not in resolution, but in durable structural remodeling marked by collagen deposition, fibroblast persistence, and ventricular stiffening (32, 60, 70–73).

Matrix cross-linking may mechanically reinforce fibroblast activation, but this RAGE-independent structural process should be distinguished from receptor-dependent inflammatory–fibrotic signaling (19, 29, 32, 60, 68–73).

### Crosstalk with toll-like receptor 4, NOD-like receptor family pyrin domain containing 3, and scavenger receptors

The potential pathogenic relevance of RAGE-dependent signaling in diabetic cardiomyopathy may be further influenced by its crosstalk with other innate immune and damage-sensing pathways, indicating that this axis operates within a broader signaling network rather than through a single linear cascade (21, 53–56, 60, 67, 68, 74, 75). Among these interacting systems, Toll-like receptor 4 is particularly important because it shares ligands, inflammatory outputs, and feed-forward properties with receptor for advanced glycation end products (53–56, 67). High-mobility group box 1, for example, can engage both receptors and thereby prolong the intensity and duration of sterile inflammatory activation in the diabetic myocardium (55, 56). In addition, recent evidence suggests that AGE-driven inflammation may also proceed through direct activation of myeloid differentiation factor 2-dependent Toll-like receptor 4 signaling, broadening the inflammatory landscape beyond classical receptor for advanced glycation end products biology alone (67). This overlap helps explain why isolated blockade of a single receptor may prove insufficient once multiple damage-associated signals have accumulated (21, 53–56, 67).

AGE–RAGE signaling also converges functionally with the NOD-like receptor family pyrin domain containing 3 inflammasome, largely through oxidative stress, inflammatory priming, and cytokine-mediated amplification (60, 68, 74). In this context, persistent receptor activation creates the redox and inflammatory conditions required for inflammasome assembly, while interleukin-1 beta and interleukin-18 release further intensify myocardial inflammation, pyroptotic injury, and fibrotic remodeling (60, 68, 74). The result is a tightly coupled inflammatory circuit in which oxidative stress, innate immune activation, and structural remodeling reinforce one another rather than acting as isolated processes (60, 68, 74). This network-level perspective is particularly relevant to diabetic cardiomyopathy because it better captures the chronic, low-grade, self-perpetuating nature of myocardial injury under diabetic conditions (21, 60, 68, 74).

In addition, AGE-related stress intersects with scavenger receptor systems, especially cluster of differentiation 36 and lectin-like oxidized low-density lipoprotein receptor 1, thereby broadening the biological impact of glycation beyond canonical receptor for advanced glycation end products signaling (64, 75, 76). Through these receptor pathways, glycation-related injury may contribute to lipid uptake, endothelial dysfunction, vascular inflammation, metabolic reprogramming, and microcirculatory stress, all of which can further aggravate myocardial vulnerability in diabetic cardiomyopathy (64, 75, 76). Overall, RAGE-related crosstalk is best interpreted as one convergence component within a wider inflammatory–oxidative network, supporting multitarget strategies without implying pathway dominance (21, 56, 60, 67, 68, 74, 75).

## Clinical relevance and biomarker potential

### Association with diastolic dysfunction, stiffness, and remodeling

Human studies support an association between tissue AGE burden and diastolic stiffness, impaired relaxation, and reduced exercise capacity (3–5, 19, 20, 77, 78). These findings do not establish that RAGE signaling is the pathway-specific cause of these phenotypes. RAGE-independent matrix cross-linking and RAGE-dependent inflammatory signaling provide complementary mechanistic hypotheses, whose relative contributions in human DCM remain uncertain (15, 19–21).

### Circulating and tissue-related biomarkers

Potential tissue-related or cumulative glycation measures include myocardial AGE/RAGE assessment in research tissue, collagen cross-linking indices, and skin autofluorescence as a noninvasive surrogate of cumulative tissue AGE burden (19, 20, 79). Myocardial tissue assessment is biologically closest to the target organ but is invasive, whereas skin autofluorescence is practical but not cardiac-specific. These measures should therefore be interpreted alongside cardiac imaging rather than as direct standalone readouts of myocardial RAGE activity.

Candidate circulating measures include N*ε*-carboxymethyl-lysine (CML), N*ε*-carboxyethyl-lysine (CEL), methylglyoxal-derived hydroimidazolone 1 (MG-H1), pentosidine, MGO-related indices, total soluble RAGE (sRAGE), endogenous secretory RAGE (esRAGE), HMGB1, and S100 calcium-binding protein A12 (S100A12) (15, 21, 24, 79, 80). Heme/hemin may reflect non-protein injury-associated ligand burden but is strongly influenced by hemolysis and systemic tissue injury. No currently available circulating marker provides a validated cardiac-specific readout of myocardial AGE or RAGE activity. Renal function, inflammatory burden, metabolic status, vascular injury, age, and diabetes stage must be considered in interpretation (15, 21, 24, 80).

A practical research framework should combine glycation/dicarbonyl markers, renal function and inflammatory context with E/e′, left atrial strain, global longitudinal strain, cardiac magnetic resonance native T1 and extracellular volume, myocardial perfusion, exercise diastolic reserve, and peak oxygen uptake (peak VO₂) (4, 5, 24, 77–79, 81, 82). Such a composite profile may support mechanistic subgroup enrichment, but it should not be presented as a validated diagnostic algorithm until externally replicated.

### Phenotypic stratification and clinical applicability

AGE-related and RAGE-dependent measures may contribute to research phenotyping within heterogeneous diabetic myocardial disease, particularly when stiffness, fibrosis, low-grade inflammation, or dicarbonyl burden is prominent (4, 5, 24, 77–81, 83). However, these features commonly overlap with obesity, hypertension, chronic kidney disease, coronary microvascular dysfunction, and cardiometabolic HFpEF. The pathway should therefore be used for subgroup enrichment within a multiorgan phenotype, not to define an isolated AGE–RAGE-driven disease entity.

At present, however, the clinical applicability of AGE–RAGE-based stratification remains more conceptual than operational (15, 21, 24). Although tissue AGE burden, circulating ligands, and receptor-related biomarkers all provide biologically meaningful signals, none has yet achieved sufficient disease specificity, standardization, or validation to function as a standalone classifier in routine practice (15, 21, 24). In addition, diabetic myocardial injury rarely reflects a single dominant mechanism; instead, AGE–RAGE-related stress often coexists with lipotoxicity, mitochondrial dysfunction, microvascular impairment, and broader cardiometabolic abnormalities (4, 5, 24, 77, 83). This complexity means that the pathway is more likely to be clinically useful as part of a multidimensional phenotyping model than as an isolated biomarker strategy (4, 5, 15, 21, 24).

Echocardiographic deformation analysis and cardiac magnetic resonance can identify stiffness-, fibrosis-, perfusion-, and strain-related phenotypes that may be useful for enrichment and endpoint selection (4, 24, 77, 78, 82). The WATCH-DM risk score may complement imaging-based enrichment in patients with type 2 diabetes and HFpEF, although it is not specific to AGE- or RAGE-related biology (84). Relevant measures include E/e′, left atrial strain, global longitudinal strain, native T1, extracellular volume, and myocardial perfusion. These measures are phenotypic endpoints rather than AGE- or RAGE-specific biomarkers, and should be integrated with biochemical and clinical data.

## Major controversies and translational barriers

### Causal contribution and contextual relevance of AGE-mediated and RAGE-dependent mechanisms in human DCM

Experimental findings support mechanistic relevance, but they do not establish pathway-specific causality in humans (3–5, 10, 15, 21, 24, 85–87). AGE accumulation and RAGE-related signaling are therefore more appropriately described as potential mediators or contributors to myocardial stress propagation, with causal weight that may vary according to phenotype, disease stage, renal function, and coexisting cardiometabolic injury (15, 21, 22, 49–52, 60, 85).

Within the heterogeneous human disease, AGE-mediated and RAGE-dependent processes may function as context-dependent stress-amplifying components rather than universal initiators (10, 15, 21, 24, 86, 87). They may contribute to persistence or progression in selected phenotypes, but their relative importance compared with lipotoxicity, mitochondrial dysfunction, microvascular injury, renal dysfunction, and systemic inflammation remains uncertain (3–5, 9–12, 24, 86, 87). This interpretation supports phenotype enrichment and combination strategies without assigning the pathway a predetermined dominant position.

### Hierarchy of current evidence

The evidence is strongest for biochemical plausibility and experimental target modulation: cellular and animal studies support AGE cross-linking, protein modification, RAGE signaling, and attenuation of selected phenotypes after pathway intervention (15, 19–22, 36–38, 50–52, 60, 85). Human evidence remains limited and predominantly associative, with the most consistent observations linking tissue AGE burden to diastolic dysfunction, stiffness, and exercise limitation (19, 20). Evidence remains insufficient for pathway-specific causality, direct myocardial target engagement, or therapeutic efficacy, and dedicated DCM randomized trials with mechanism-matched endpoints are lacking (10, 24).

### Interpretive limitations of circulating AGE–RAGE-related biomarkers

Circulating AGE species, sRAGE isoforms, and injury-associated ligands should be interpreted as composite systemic cardiometabolic signals influenced by renal clearance, vascular dysfunction, inflammation, age, and disease stage (15, 24, 39–41, 88–90). They may support research stratification when integrated with imaging and functional assessment, but they are not currently validated as standalone surrogates of myocardial biology or independent treatment-selection tools (4, 5, 21, 24).

### Barriers to translating preclinical AGE–RAGE-targeted strategies into diabetic cardiomyopathy-specific therapies

Preclinical intervention studies demonstrate experimental modifiability of selected AGE- or RAGE-related processes, but this should not be equated with clinical therapeutic actionability (36–38, 50–52, 60). Available models often simplify human DCM and do not fully reproduce aging, obesity, hypertension, renal dysfunction, microvascular disease, multimorbidity, or established fibrosis (4, 10, 24, 77, 78, 91, 92). Translation therefore requires disease definitions, enrichment strategies, target-engagement measures, and endpoints that match the proposed mechanism.

### Key barriers to clinical implementation

Future studies should prespecify mechanism-based eligibility and stratification variables (for example, AGE/dicarbonyl burden, estimated glomerular filtration rate (eGFR), obesity, hypertension, HFpEF phenotype, fibrosis, and disease stage), demonstrate target engagement, and select endpoints matched to the intervention (24, 82, 93–96). Early-phase studies should prioritize biochemical target engagement, E/e′, left atrial strain, global longitudinal strain, cardiac magnetic resonance native T1/extracellular volume, perfusion, peak VO₂, and exercise diastolic reserve. Hospitalization and cardiovascular outcomes are more appropriate for later, adequately powered trials.

### Proposed framework for phenotype-enriched AGE- or RAGE-targeted trials

A future trial should enroll patients with type 2 diabetes and objective evidence of subclinical or overt myocardial dysfunction that is not fully explained by obstructive coronary disease or significant valvular disease. Hypertension, obesity, and chronic kidney disease should be recorded and stratified rather than automatically excluded, because they are integral to the real-world cardiometabolic phenotype. Mechanistic enrichment may include elevated AGE/dicarbonyl burden, increased skin autofluorescence, abnormal E/e′ or strain, or cardiac magnetic resonance evidence of diffuse fibrosis. Randomization should be stratified by eGFR, obesity, hypertension, HFpEF phenotype, diabetes duration, and baseline fibrosis. Endpoints should be prespecified by phase and mechanism. Target-engagement endpoints should match the intervention, including MGO, MG-H1, or GLO1 activity for dicarbonyl-lowering strategies and candidate RAGE/DIAPH1-related pharmacodynamic measures for receptor-directed interventions. Mechanism-matched intermediate cardiac efficacy endpoints may include E/e′, left atrial strain, global longitudinal strain, cardiac magnetic resonance native T1/extracellular volume, myocardial perfusion, endothelial function, exercise diastolic reserve, and peak VO₂. Renal and vascular measures, including eGFR slope and albuminuria, may serve as secondary endpoints in multiorgan phenotypes, whereas heart failure hospitalization and cardiovascular events should be reserved for later, adequately powered trials after biological activity has been demonstrated (24, 82, 93, 95, 96).

## Therapeutic landscape targeting AGE-mediated injury and RAGE-dependent signaling

### Upstream reduction of AGE formation and carbonyl stress

Current clinical practice primarily reduces the upstream metabolic conditions that favor AGE and dicarbonyl formation through glycemic optimization, weight management, exercise, renal preservation, and evidence-based cardiometabolic therapy (6, 7, 10, 15, 18, 24). Sodium-glucose cotransporter 2 (SGLT2) inhibitors and glucagon-like peptide-1 (GLP-1) receptor agonists have established cardiovascular or cardiorenal benefits and may indirectly reduce the metabolic substrate for glycation, but their direct effects on myocardial AGE accumulation, RAGE activation, or DCM-specific target engagement remain incompletely defined (6, 7, 10, 24).

### Interruption of matrix cross-linking and glycation chemistry

Intermediate strategies include carbonyl scavenging, inhibition of AGE formation, and enhancement of GLO1-dependent detoxification of MGO and related dicarbonyls, including glyoxal and 3-DG (18, 30, 31, 97). These approaches may reduce both direct intracellular dicarbonyl modification and subsequent AGE formation, but evidence for myocardial target engagement in humans is lacking. AGE cross-link breakers such as alagebrium directly address the RAGE-independent structural component and improve selected preclinical phenotypes (36–38); however, DCM-specific clinical efficacy and safety have not been established (24, 36–38).

### Direct targeting of RAGE-related signaling

Direct RAGE-related strategies include extracellular ligand blockade or decoy approaches and intracellular disruption of the RAGE–DIAPH1 interface (49–52, 98–104). The RAGE–DIAPH1 interaction is a candidate intracellular signaling interface, but evidence is derived mainly from cellular, vascular, inflammatory, atherosclerotic, septic, or ischemia/reperfusion settings rather than DCM-specific human studies (49–52, 98–105). These approaches should therefore be described as early-stage experimental strategies, with unresolved cardiac selectivity, pharmacokinetics, long-term safety, and target-engagement requirements.

### Multitarget and phenotype-guided therapeutic strategies

Because DCM is embedded within a broader cardiovascular–kidney–metabolic phenotype, AGE- or RAGE-directed treatment should be evaluated as an adjunct to contemporary cardiometabolic care, not as an alternative or universal monotherapy (15, 24, 106–108). Patients with greater glycation/dicarbonyl burden, stiffness, fibrosis, or inflammatory activation may be reasonable enrichment candidates, but this remains a testable hypothesis rather than an established treatment-selection rule. Combination and sequencing strategies should be guided by dominant organ involvement, disease stage, and mechanism-matched endpoints. These therapeutic strategies and their translational limitations are summarized in Table 3 (15, 21, 24, 106–108).

**Table 3:** Therapeutic strategies targeting AGE formation, RAGE-independent matrix injury, or RAGE-dependent signaling in diabetic cardiomyopathy and their translational limitations.

Intervention level	Representative approaches	Main mechanistic target	Current evidence/major translational limitations	Ref.
Upstream: metabolic optimization and carbonyl stress reduction	Glycemic and lipid control; weight management; exercise; dietary AGE restriction; SGLT2 inhibitors; GLP-1 receptor agonists	Reduces substrate excess and dicarbonyl stress, thereby restraining expansion of the systemic AGE pool	Clinical cardiometabolic benefit is established, but direct anti-glycation effects in DCM remain largely inferential	(6, 7, 18, 24)
Midstream: dicarbonyl and AGE-formation reduction	Carbonyl scavenging; glyoxalase enhancement; pyridoxamine	Reduces reactive dicarbonyl burden, intracellular protein modification, and subsequent AGE formation	Mostly preclinical; durability, safety, and DCM-specific clinical validation remain insufficient	(18, 31, 97)
Midstream: RAGE-independent matrix cross-link modulation	AGE cross-link breakers such as alagebrium	Targets established collagen cross-links and myocardial stiffness independently of RAGE signaling	Preclinical evidence suggests improved ventricular compliance, but durability, safety, and DCM-specific clinical validation remain insufficient	(24, 36–38)
Downstream: direct RAGE-related inhibition	sRAGE; peptoids; aptamers; small-molecule antagonists; RAGE–DIAPH1 inhibitors	Blocks ligand engagement or intracellular signal propagation, thereby suppressing inflammatory and oxidative amplification	Promising but early-stage; long-term safety, tissue selectivity, pharmacokinetics, and DCM-specific feasibility remain unclear	(21, 49–52, 98–101, 105)
Multitarget and phenotype-guided strategies	Combination approaches integrating cardiometabolic therapy with anti-inflammatory, antifibrotic, or inflammasome-oriented modulation; phenotype-enriched enrollment	Targets parallel nodes across the metabolism–inflammation–fibrosis axis and aligns therapy with disease stage and phenotype	Conceptually strong but not standardized; requires biomarker/imaging enrichment and dedicated DCM trials	(4, 15, 24, 60, 68, 69, 77, 78)

AGE, advanced glycation end product; RAGE, receptor for advanced glycation end products; DCM, diabetic cardiomyopathy; sRAGE, soluble receptor for advanced glycation end products; DIAPH1, diaphanous-related formin 1; SGLT2, sodium-glucose cotransporter 2; GLP-1, glucagon-like peptide-1.

The table separates upstream metabolic optimization, dicarbonyl/AGE-formation control, RAGE-independent matrix-directed strategies, and receptor-dependent interventions. Most pathway-specific evidence is preclinical, and no strategy has established DCM-specific outcome benefit. Candidate clinical endpoints should be matched to the proposed mechanism and evaluated in phenotype-enriched populations.

## Discussion

The present synthesis supports AGE-mediated injury and RAGE-dependent signaling as biologically plausible, context-dependent contributors to diabetic myocardial remodeling rather than as universal upstream determinants of DCM (15, 19–22, 51, 52, 60). RAGE-independent matrix cross-linking, intracellular dicarbonyl-related protein modification, and RAGE-dependent multiligand signaling are distinct but interacting processes. DIAPH1 provides a candidate intracellular interface for RAGE signaling, but its myocardial relevance in human DCM remains predominantly supported by preclinical evidence. These mechanisms operate within a broader network of metabolic inflexibility, lipotoxicity, mitochondrial stress, inflammation, fibrosis, microvascular dysfunction, renal disease, and aging (3, 4, 10–12).

Human data most consistently associate tissue AGE burden with a stiffness-dominant phenotype characterized by impaired relaxation and reduced exercise capacity (3, 4, 15, 19–22). This supports the hypothesis that patients with greater glycation/dicarbonyl burden, fibrosis, or inflammatory activation may be enriched for AGE- or RAGE-related mechanisms. However, no validated biomarker panel currently identifies such a subgroup with adequate cardiac specificity, and this concept should be tested prospectively rather than presented as established clinical stratification.

The translational gap reflects a clear evidence hierarchy: biochemical and preclinical support is substantial, human association is moderate, and DCM-specific causal, target-engagement, and therapeutic evidence is weak (10, 24, 31, 60, 97, 109). Additional experimental studies implicate AGE/ROS-related ferroptotic stress in diabetic complications and HMGB1/TLR4/IL-33-mediated cardiomyocyte–fibroblast crosstalk in murine DCM (110, 111). Findings from atherosclerosis, sepsis, ischemia/reperfusion, or other diabetic complications provide mechanistic context but should not be treated as direct evidence of efficacy in DCM. Multimorbidity, advanced fibrosis, and disease-stage differences further limit extrapolation from prevention-oriented models.

Future progress requires phenotype-enriched, mechanism-matched studies that first demonstrate target engagement and improvement in relevant biochemical, imaging, or functional endpoints before advancing to outcome trials (3, 4, 10–12, 15, 19–22, 24). AGE- or RAGE-directed interventions should be embedded within contemporary cardiometabolic care and evaluated according to whether they modify the specific structural or signaling process they are designed to target. The key question is not whether the pathway has “failed,” but which component, patient subgroup, disease stage, and endpoint are appropriate for clinical testing.

## Conclusion

AGE-mediated matrix modification, intracellular dicarbonyl-related protein injury, and RAGE-dependent multiligand signaling represent distinct but interacting, biologically plausible components of diabetic myocardial remodeling. They may contribute to stiffness, impaired relaxation, inflammatory activation, endothelial dysfunction, and fibrosis, but current human evidence remains predominantly associative and does not establish a universally dominant or pathway-specific causal role.

Clinical translation is not yet established. No circulating biomarker provides a validated cardiac-specific readout, direct myocardial target engagement is rarely demonstrated, and DCM-specific therapeutic outcome trials are lacking. Future research should prioritize multimodal phenotype enrichment, mechanism-matched endpoints, disease-stage selection, and integration with contemporary cardiometabolic therapy.

## References

[1] Rubler S Dlugash J Yuceoglu YZ Kumral T Branwood AW Grishman A. New type of cardiomyopathy associated with diabetic glomerulosclerosis. Am J Cardiol. (1972) 30:595–602. 10.1016/0002-9149(72)90595-4 4263660

[2] Jia G Hill MA Sowers JR. Diabetic cardiomyopathy: an update of mechanisms contributing to this clinical entity. Circ Res. (2018) 122:624–38. 10.1161/CIRCRESAHA.117.311586 29449364 PMC5819359

[3] Lee MMY McMurray JJV Lorenzo-Almorós A Kristensen SL Sattar N Jhund PS. Diabetic cardiomyopathy. Heart. (2019) 105:337–45. 10.1136/heartjnl-2016-310342 30337334

[4] Marwick TH Ritchie R Shaw JE Kaye D. Implications of underlying mechanisms for the recognition and management of diabetic cardiomyopathy. J Am Coll Cardiol. (2018) 71:339–51. 10.1016/j.jacc.2017.11.019 29348027

[5] Zhao X Liu S Wang X Chen Y Pang P Yang Q. Diabetic cardiomyopathy: clinical phenotype and practice. Front Endocrinol (Lausanne). (2022) 13:1032268. 10.3389/fendo.2022.1032268 36568097 PMC9767955

[6] Mann C Braunwald E Zelniker TA. Diabetic cardiomyopathy revisited: the interplay between diabetes and heart failure. Int J Cardiol. (2025) 438:133554. 10.1016/j.ijcard.2025.133554 40571130

[7] Balakrishnan B Hariharapura RC Nayak V Mahyoub MA Raghu Chandrashekar H. Hyperglycaemia to heart failure: molecular pathophysiology, clinical manifestations, and novel therapeutic targets for diabetic cardiomyopathy. Arch Physiol Biochem. (2026) 132:241–54. 10.1080/13813455.2025.2567336 41066522

[8] De Geest B Mishra M. Role of oxidative stress in diabetic cardiomyopathy. Antioxidants. (2022) 11:784. 10.3390/antiox11040784 35453469 PMC9030255

[9] Ritchie RH Abel ED. Basic mechanisms of diabetic heart disease. Circ Res. (2020) 126:1501–25. 10.1161/CIRCRESAHA.120.315913 32437308 PMC7251974

[10] Tan Y Zhang Z Zheng C Wintergerst KA Keller BB Cai L. Mechanisms of diabetic cardiomyopathy and potential therapeutic strategies: preclinical and clinical evidence. Nat Rev Cardiol. (2020) 17:585–607. 10.1038/s41569-020-0339-2 32080423 PMC7849055

[11] Heather LC Gopal K Srnic N Ussher JR. Redefining diabetic cardiomyopathy: perturbations in substrate metabolism at the heart of its pathology. Diabetes. (2024) 73:659–70. 10.2337/dbi23-0019 38387045 PMC11043056

[12] Ye W Han K Xie M Li S Chen G Wang Y. Mitochondrial energy metabolism in diabetic cardiomyopathy: physiological adaption, pathogenesis, and therapeutic targets. Chin Med J (Engl). (2024) 137:936–48. 10.1097/CM9.0000000000003075 38527931 PMC11046025

[13] Qaed E Aldahmash W Mahyoub MA. Advanced glycation end products (AGEs) and their role in diabetes mellitus and related complications: mechanisms and therapeutic insights. Glycoconj J. (2025) 42:209–23. 10.1007/s10719-025-10194-x 40982076

[14] Wasim R Mahmood T Siddiqui MH Ahsan F Shamim A Singh A. Aftermath of AGE-RAGE cascade in the pathophysiology of cardiovascular ailments. Life Sci. (2022) 307:120860. 10.1016/j.lfs.2022.120860 35940220

[15] Mota KO De Vasconcelos CML Kirshenbaum LA Dhalla NS. The role of advanced glycation end-products in the pathophysiology and pharmacotherapy of cardiovascular disease. Int J Mol Sci. (2025) 26:7311. 10.3390/ijms26157311 40806443 PMC12347567

[16] Goldin A Beckman JA Schmidt AM Creager MA. Advanced glycation end products: sparking the development of diabetic vascular injury. Circulation. (2006) 114:597–605. 10.1161/CIRCULATIONAHA.106.621854 16894049

[17] Ott C Jacobs K Haucke E Navarrete Santos A Grune T Simm A. Role of advanced glycation end products in cellular signaling. Redox Biol. (2014) 2:411–29. 10.1016/j.redox.2013.12.016 24624331 PMC3949097

[18] Song H Ma H Shi J Liu Y Kan C Hou N. Optimizing glycation control in diabetes: an integrated approach for inhibiting nonenzymatic glycation reactions of biological macromolecules. Int J Biol Macromol. (2023) 243:125148. 10.1016/j.ijbiomac.2023.125148 37268079

[19] van Heerebeek L Hamdani N Handoko ML Falcao-Pires I Musters R Kupreishvili K. Diastolic stiffness of the failing diabetic heart: importance of fibrosis, advanced glycation end products, and myocyte resting tension. Circulation. (2008) 117:43–51. 18071071 10.1161/CIRCULATIONAHA.107.728550

[20] Willemsen S Hartog JWL Hummel YM Van Ruijven MHI Van Der Horst ICC Van Veldhuisen DJ. Tissue advanced glycation end products are associated with diastolic function and aerobic exercise capacity in diabetic heart failure patients. Eur J Heart Fail. (2011) 13:76–82. 10.1093/eurjhf/hfq168 20861128

[21] Egaña-Gorroño L López-Díez R Yepuri G Ramirez LS Reverdatto S Gugger PF. Receptor for advanced glycation end products and mechanisms and therapeutic opportunities in diabetes and cardiovascular disease: insights from human subjects and animal models. Front Cardiovasc Med. (2020) 7:37. 10.3389/fcvm.2020.00037 32211423 PMC7076074

[22] Zhou M Zhang Y Shi L Li L Zhang D Gong Z. Activation and modulation of the AGEs-RAGE axis: implications for inflammatory pathologies and therapeutic interventions—a review. Pharmacol Res. (2024) 206:107282. 10.1016/j.phrs.2024.107282 38914383

[23] Delligatti CE Kirk JA. Glycation in the cardiomyocyte. Vitam Horm. (2024) 125:47–88. 10.1016/bs.vh.2024.04.005 38997172 PMC11578284

[24] Khattab E Kyriakou M Leonidou E Sokratous S Mouzarou A Myrianthefs MM. Critical appraisal of pharmaceutical therapy in diabetic cardiomyopathy—challenges and prospectives. Pharmaceuticals. (2025) 18:134. 10.3390/ph18010134 39861195 PMC11768626

[25] Brownlee M. Biochemistry and molecular cell biology of diabetic complications. Nature. (2001) 414:813–20. 10.1038/414813a 11742414

[26] Singh R Barden A Mori T Beilin L. Advanced glycation end-products: a review. Diabetologia. (2001) 44:129–46. 10.1007/s001250051591 11270668

[27] Kuzan A. Toxicity of advanced glycation end products (review). Biomed Rep. (2021) 14:46. 10.3892/br.2021.1422 33786175 PMC7995243

[28] Thorpe SR Baynes JW. Role of the maillard reaction in diabetes mellitus and diseases of aging. Drugs Aging. (1996) 9:69–77. 10.2165/00002512-199609020-00001 8820792

[29] Neff LS Bradshaw AD. Cross your heart? Collagen cross-links in cardiac health and disease. Cell Signal. (2021) 79:109889. 10.1016/j.cellsig.2020.109889 33347984 PMC8830414

[30] Rabbani N Thornalley PJ. Methylglyoxal, glyoxalase 1 and the dicarbonyl proteome. Amino Acids. (2012) 42:1133–42. 10.1007/s00726-010-0783-0 20963454

[31] Brouwers O Vos-Houben J Niessen P Miyata T Nieuwenhoven F Janssen B. Mild oxidative damage in the diabetic rat heart is attenuated by glyoxalase-1 overexpression. Int J Mol Sci. (2013) 14:15724–39. 10.3390/ijms140815724 23899787 PMC3759882

[32] Liang B Zhou Z Yang Z Liu J Zhang L He J. AGEs-RAGE axis mediates myocardial fibrosis via activation of cardiac fibroblasts induced by autophagy in heart failure. Exp Physiol. (2022) 107:879–91. 10.1113/EP090042 35598104

[33] Yan D Luo X Li Y Liu W Deng J Zheng N. Effects of advanced glycation end products on calcium handling in cardiomyocytes. Cardiology. (2014) 129:75–83. 10.1159/000364779 25138529

[34] Petrova R Yamamoto Y Muraki K Yonekura H Sakurai S Watanabe T. Advanced glycation endproduct-induced calcium handling impairment in mouse cardiac myocytes. J Mol Cell Cardiol. (2002) 34:1425–31. 10.1006/jmcc.2002.2084 12393002

[35] Hegab Z Mohamed TMA Stafford N Mamas M Cartwright EJ Oceandy D. Advanced glycation end products reduce the calcium transient in cardiomyocytes by increasing production of reactive oxygen species and nitric oxide. FEBS Open Bio. (2017) 7:1672–85. 10.1002/2211-5463.12284 29123976 PMC5666397

[36] Candido R Forbes JM Thomas MC Thallas V Dean RG Burns WC. A breaker of advanced glycation end products attenuates diabetes-induced myocardial structural changes. Circ Res. (2003) 92:785–92. 10.1161/01.RES.0000065620.39919.20 12623881

[37] Liu J Masurekar MR Vatner DE Jyothirmayi GN Regan TJ Vatner SF. Glycation end-product cross-link breaker reduces collagen and improves cardiac function in aging diabetic heart. Am J Physiol Heart Circ Physiol. (2003) 285:H2587–91. 10.1152/ajpheart.00516.2003 12946933

[38] Kranstuber AL Del Rio C Biesiadecki BJ Hamlin RL Ottobre J Gyorke S. Advanced glycation end product cross-link breaker attenuates diabetes-induced cardiac dysfunction by improving sarcoplasmic reticulum calcium handling. Front Physiol. (2012) 3:292. 10.3389/fphys.2012.00292 22934044 PMC3429064

[39] Vlassara H Palace MR Diabetes and advanced glycation end products. J Intern Med. (2002) 251:87–101. 10.1046/j.1365-2796.2002.00932.x 11905595

[40] Vlassara H Uribarri J. Advanced glycation end products (AGE) and diabetes: cause, effect, or both? Curr Diab Rep. (2014) 14:453. 10.1007/s11892-013-0453-2 24292971 PMC3903318

[41] Wu XQ Zhang DD Wang YN Tan YQ Yu XY Zhao YY. AGE/RAGE in diabetic kidney disease and ageing kidney. Free Radic Biol Med. (2021) 171:260–71. 10.1016/j.freeradbiomed.2021.05.025 34019934

[42] Cai W He JC Zhu L Lu C Vlassara H. Advanced glycation end product receptor 1 suppresses cell oxidant stress and activation signaling via EGF receptor. Proc Natl Acad Sci U S A. (2006) 103:13801–6. 10.1073/pnas.0600362103 16954185 PMC1564251

[43] Neeper M Schmidt AM Brett J Yan SD Wang F Pan YC. Cloning and expression of a cell surface receptor for advanced glycosylation end products of proteins. J Biol Chem. (1992) 267:14998–5004. 10.1016/S0021-9258(18)42138-2 1378843

[44] Indurthi VSK Jensen JL Leclerc E Sinha S Colbert CL Vetter SW. The trp triad within the V-domain of the receptor for advanced glycation end products modulates folding, stability and ligand binding. Biosci Rep. (2020) 40:BSR20193360. 10.1042/BSR20193360 31912881 PMC6997106

[45] Hudson BI Kalea AZ Del Mar Arriero M Harja E Boulanger E D'Agati V. Interaction of the RAGE cytoplasmic domain with diaphanous-1 is required for ligand-stimulated cellular migration through activation of Rac1 and Cdc42. J Biol Chem. (2008) 283:34457–68. 10.1074/jbc.M801465200 18922799 PMC2590709

[46] Brett J Schmidt AM Yan SD Zou YS Weidman E Pinsky D. Survey of the distribution of a newly characterized receptor for advanced glycation end products in tissues. Am J Pathol. (1993) 143:1699–712. 8256857 PMC1887265

[47] Schmidt AM Yan SD Yan SF Stern DM. The multiligand receptor RAGE as a progression factor amplifying immune and inflammatory responses. J Clin Invest. (2001) 108:949–55. 10.1172/JCI14002 11581294 PMC200958

[48] Dong W Yang X Li X Wei S An C Zhang J. Investigation of N-glycan functions in receptor for advanced glycation end products V domain through chemical glycoprotein synthesis. J Am Chem Soc. (2024) 146:18270–80. 10.1021/jacs.4c01413 38917169

[49] Theophall GG Manigrasso MB Nazarian P Premo A Reverdatto S Yepuri G. RAGE-mediated activation of the formin DIAPH1 and human macrophage inflammation are inhibited by a small molecule antagonist. Cell Chem Biol. (2025) 32:1221–1234.e8. 10.1016/j.chembiol.2025.09.004 41038162 PMC12614354

[50] Manigrasso MB Rabbani P Egaña-Gorroño L Quadri N Frye L Zhou B. Small-molecule antagonism of the interaction of the RAGE cytoplasmic domain with DIAPH1 reduces diabetic complications in mice. Sci Transl Med. (2021) 13:eabf7084. 10.1126/scitranslmed.abf7084 34818060 PMC8669775

[51] Ramasamy R Shekhtman A Schmidt AM. The RAGE/DIAPH1 signaling axis and implications for the pathogenesis of diabetic complications. Int J Mol Sci. (2022) 23:4579. 10.3390/ijms23094579 35562970 PMC9102165

[52] Yepuri G Hasan SN Kumar V Manigrasso MB Theophall G Shekhtman A. Mechanistic underpinnings of AGEs-RAGE via DIAPH1 in ischemic, diabetic, and failing hearts. Am J Physiol Heart Circ Physiol. (2025) 328:H1026–37. 10.1152/ajpheart.00685.2024 PMC12354004 40132210

[53] Hudson BI Lippman ME. Targeting RAGE signaling in inflammatory disease. Annu Rev Med. (2018) 69:349–64. 10.1146/annurev-med-041316-085215 29106804

[54] Cross K Vetter SW Alam Y Hasan MZ Nath AD Leclerc E. Role of the receptor for advanced glycation end products and its ligands in inflammatory responses. Biomolecules. (2024) 14:1550. 39766257 10.3390/biom14121550 PMC11673996

[55] Zhong H Li X Zhou S Jiang P Liu X Ouyang M. Interplay between RAGE and TLR4 regulates HMGB1-induced inflammation by promoting cell surface expression of RAGE and TLR4. J Immunol. (2020) 205:767–75. 10.4049/jimmunol.1900860 32580932

[56] Prantner D Nallar S Vogel SN. The role of RAGE in host pathology and crosstalk between RAGE and TLR4 in innate immune signal transduction pathways. FASEB J. (2020) 34:15659–74. 33131091 10.1096/fj.202002136R PMC8121140

[57] Gellen B Thorin-Trescases N Thorin E Gand E Ragot S Montaigne D. Increased serum S100A12 levels are associated with higher risk of acute heart failure in patients with type 2 diabetes. ESC Heart Fail. (2022) 9:3909–19. 10.1002/ehf2.14036 36637406 PMC9773733

[58] Yepuri G Shekhtman A Schmidt AM Ramasamy R. Heme & RAGE: a new opportunistic relationship? FEBS J. (2021) 288:3424–7. 10.1111/febs.15723 33565264

[59] May O Yatime L Merle NS Delguste F Howsam M Daugan MV. The receptor for advanced glycation end products is a sensor for cell-free heme. FEBS J. (2021) 288:3448–64. 10.1111/febs.15667 33314778

[60] Hashiesh HM Azimullah S Nagoor Meeran MF Saraswathiamma D Arunachalam S Jha NK. Cannabinoid 2 receptor activation protects against diabetic cardiomyopathy through inhibition of AGE/RAGE-induced oxidative stress, fibrosis, and inflammasome activation. J Pharmacol Exp Ther. (2024) 391:241–57. 10.1124/jpet.123.002037 38955492

[61] Yang DR Wang MY Zhang CL Wang Y. Endothelial dysfunction in vascular complications of diabetes: a comprehensive review of mechanisms and implications. Front Endocrinol. (2024) 15:1359255. 10.3389/fendo.2024.1359255 PMC11026568 38645427

[62] Salvatore T Galiero R Caturano A Vetrano E Loffredo G Rinaldi L. Coronary microvascular dysfunction in diabetes mellitus: pathogenetic mechanisms and potential therapeutic options. Biomedicines. (2022) 10:2274. 10.3390/biomedicines10092274 36140374 PMC9496134

[63] Horton WB Barrett EJ. Microvascular dysfunction in diabetes mellitus and cardiometabolic disease. Endocr Rev. (2021) 42:29–55. 10.1210/endrev/bnaa025 33125468 PMC7846151

[64] Yamazaki Y Wake H Nishinaka T Hatipoglu OF Liu K Watanabe M. Involvement of multiple scavenger receptors in advanced glycation end product-induced vessel tube formation in endothelial cells. Exp Cell Res. (2021) 408:112857. 10.1016/j.yexcr.2021.112857 34600900

[65] Musigk N Suwalski P Golpour A Fairweather DL Klingel K Martin P. The inflammatory spectrum of cardiomyopathies. Front Cardiovasc Med. (2024) 11:1251780. 10.3389/fcvm.2024.1251780 38464847 PMC10921946

[66] Bellemare M Bourcier L Iglesies-Grau J Boulet J O'Meara E Bouabdallaoui N. Mechanisms of diabetic cardiomyopathy: focus on inflammation. Diabetes Obes Metab. (2025) 27(5):2326–38. 10.1111/dom.16242 39930551 PMC11964996

[67] Wang Y Luo W Han J Khan ZA Fang Q Jin Y. MD2 Activation by direct AGE interaction drives inflammatory diabetic cardiomyopathy. Nat Commun. (2020) 11:2148. 10.1038/s41467-020-15978-3 32358497 PMC7195432

[68] Sun Q Guo W Wang L Mou H. NLRP3 Inflammasome mechanism and therapeutic targets in diabetic cardiomyopathy. Gac Med Mex. (2023) 159:255–61. 10.24875/GMM.22000403 37494725

[69] Ramesh P Yeo JL Brady EM McCann GP. Role of inflammation in diabetic cardiomyopathy. Ther Adv Endocrinol Metab. (2022) 13:20420188221083530. 10.1177/20420188221083530 35308180 PMC8928358

[70] Cheng Y Wang Y Yin R Xu Y Zhang L Zhang Y. Central role of cardiac fibroblasts in myocardial fibrosis of diabetic cardiomyopathy. Front Endocrinol. (2023) 14:1162754. 10.3389/fendo.2023.1162754 PMC10102655 37065745

[71] Levick SP Widiapradja A. The diabetic cardiac fibroblast: mechanisms underlying phenotype and function. Int J Mol Sci. (2020) 21:970. 10.3390/ijms21030970 32024054 PMC7036958

[72] Li JH Huang XR Zhu HJ Oldfield M Cooper M Truong LD. Advanced glycation end products activate Smad signaling via transforming growth factor-beta-dependent and independent mechanisms: implications for diabetic renal and vascular disease. FASEB J. (2004) 18:176–8. 10.1096/fj.02-1117fje 12709399

[73] Meng XM Nikolic-Paterson DJ Lan HY. TGF-*β*: the master regulator of fibrosis. Nat Rev Nephrol. (2016) 12:325–38. 10.1038/nrneph.2016.48 27108839

[74] Toldo S Abbate A. The role of the NLRP3 inflammasome and pyroptosis in cardiovascular diseases. Nat Rev Cardiol. (2024) 21:219–37. 37923829 10.1038/s41569-023-00946-3 PMC11550901

[75] Zhang X Fan J Li H Chen C Wang Y. CD36 Signaling in diabetic cardiomyopathy. Aging Dis. (2021) 12:826–40. 10.14336/AD.2020.1217 34094645 PMC8139204

[76] Wang ZQ Yao HP Sun Z. N*ε*-(carboxymethyl)lysine promotes lipid uptake of macrophage via cluster of differentiation 36 and receptor for advanced glycation end products. World J Diabetes. (2023) 14:222–33. 10.4239/wjd.v14.i3.222 37035231 PMC10075039

[77] Gulsin GS Athithan L McCann GP. Diabetic cardiomyopathy: prevalence, determinants and potential treatments. Ther Adv Endocrinol Metab. (2019) 10:2042018819834869. 10.1177/2042018819834869 30944723 PMC6437329

[78] Seferović PM Paulus WJ. Clinical diabetic cardiomyopathy: a two-faced disease with restrictive and dilated phenotypes. Eur Heart J. (2015) 36:1718–27. 10.1093/eurheartj/ehv134 25888006

[79] Ianoș RD Cozma A Lucaciu RL Hangan AC Negrean V Mercea DC. Role of circulating biomarkers in diabetic cardiomyopathy. Biomedicines. (2024) 12:2153. 10.3390/biomedicines12092153 39335666 PMC11428922

[80] Chen Q Liu L Ke W Li X Xiao H Li Y. Association between the soluble receptor for advanced glycation end products and diabetes mellitus: systematic review and meta-analysis. BMC Endocr Disord. (2024) 24:232. 10.1186/s12902-024-01759-2 39472884 PMC11523792

[81] Huang S Li X Zhang L Sun R Liu H Zhang X. Cardiac MRI in diabetic cardiomyopathy: translating imaging biomarkers into clinical practice. Cardiovasc Diabetol. (2026) 25:4. 10.1186/s12933-025-03019-6 PMC12781312 41345656

[82] Chen H Mankad S. The role of multimodality imaging in diabetic cardiomyopathy: a brief review. Front Endocrinol (Lausanne). (2024) 15:1405031. 10.3389/fendo.2024.1405031 39764242 PMC11700833

[83] Gorica E Paneni F. Cardiometabolic HFpEF with focus on type 2 diabetes mellitus. Cardiovasc Diabetol. (2025) 24:390. 10.1186/s12933-025-02955-7 41068770 PMC12513086

[84] Iwakura K Onishi T Okamura A Koyama Y Tanaka N Okada M. The WATCH-DM risk score estimates clinical outcomes in type 2 diabetic patients with heart failure with preserved ejection fraction. Sci Rep. (2024) 14:1746. 10.1038/s41598-024-52101-8 38243047 PMC10798943

[85] Bugger H Abel ED. Molecular mechanisms of diabetic cardiomyopathy. Diabetologia. (2014) 57:660–71. 10.1007/s00125-014-3171-6 24477973 PMC3969857

[86] Kenny HC Abel ED. Heart failure in type 2 diabetes mellitus. Circ Res. (2019) 124:121–41. 10.1161/CIRCRESAHA.118.311371 30605420 PMC6447311

[87] Paulus WJ Tschöpe C. A novel paradigm for heart failure with preserved ejection fraction: comorbidities drive myocardial dysfunction and remodeling through coronary microvascular endothelial inflammation. J Am Coll Cardiol. (2013) 62:263–71. 10.1016/j.jacc.2013.02.092 23684677

[88] Steenbeke M Speeckaert R Desmedt S Glorieux G Delanghe JR Speeckaert MM. The role of advanced glycation end products and its soluble receptor in kidney diseases. Int J Mol Sci. (2022) 23:3439. 10.3390/ijms23073439 35408796 PMC8998875

[89] Adeshara K Lithovius R Mutter S Harjutsalo V Lehto M Groop P-H. Soluble RAGE further stratifies risk of coronary artery and end-stage kidney disease in high-risk individuals with type 1 diabetes and treatment-resistant hypertension. Cardiovasc Diabetol. (2026) 25:13. 10.1186/s12933-025-03017-8 PMC12805710 41387868

[90] Henkens MTHM Remmelzwaal S Robinson EL Van Ballegooijen AJ Barandiarán Aizpurua A Verdonschot JAJ. Risk of bias in studies investigating novel diagnostic biomarkers for heart failure with preserved ejection fraction: a systematic review. Eur J Heart Fail. (2020) 22:1586–97. 10.1002/ejhf.1944 32592317 PMC7689920

[91] Smati H Qadeer YK Rodriguez M Moras E Fonarow GC Isaacs SD. Diabetic cardiomyopathy: what clinicians should know. Am J Med. (2025) 138(3):387–95. 10.1016/j.amjmed.2024.10.026 39505128

[92] Balakrishnan B Somashekara DM Nayak V Hariharapura RC. Diabetic cardiomyopathy: a comprehensive review of diagnosis, management, and future directions. Diabetol Metab Syndr. (2025) 17:422. 10.1186/s13098-025-01986-0 41214779 PMC12599074

[93] Duan XP Qin BD Jiao XD Liu K Wang Z Zang YS. New clinical trial design in precision medicine: discovery, development and direction. Signal Transduct Target Ther. (2024) 9:57. 10.1038/s41392-024-01760-0 38438349 PMC10912713

[94] He Q Lai Z Zhai Z Zou B Shi Y Feng C. Advances of research in diabetic cardiomyopathy: diagnosis and the emerging application of sequencing. Front Cardiovasc Med. (2025) 11:1501735. 10.3389/fcvm.2024.1501735 39872882 PMC11769946

[95] Campbell P Rutten FH Lee MMY Hawkins NM Petrie MC. Heart failure with preserved ejection fraction: everything the clinician needs to know. Lancet. (2024) 403:1083–92. 10.1016/S0140-6736(23)02756-3 38367642

[96] Reddy YNV Kaye DM Handoko ML Van De Bovenkamp AA Tedford RJ Keck C. Diagnosis of heart failure with preserved ejection fraction among patients with unexplained dyspnea. JAMA Cardiol. (2022) 7:891–9. 10.1001/jamacardio.2022.1916 35830183 PMC9280610

[97] Hanssen NMJ Tikellis C Pickering RJ Dragoljevic D Lee MKS Block T. Pyridoxamine prevents increased atherosclerosis by intermittent methylglyoxal spikes in the aortic arches of ApoE-/- mice. Biomed Pharmacother. (2023) 158:114211. 10.1016/j.biopha.2022.114211 36916437

[98] Waugh ML Wolf LM Turner JP Phillips LN Servoss SL Moss MA. Modulating the RAGE-induced inflammatory response: peptoids as RAGE antagonists. Chembiochem. (2023) 24:e202300503. 10.1002/cbic.202300503 37679300 PMC10711691

[99] Koga Y Sotokawauchi A Higashimoto Y Nishino Y Hashizume N Kakuma T. DNA-aptamer raised against receptor for advanced glycation end products improves survival rate in septic mice. Oxid Med Cell Longev. (2021) 2021:9932311. 10.1155/2021/9932311 34413930 PMC8369179

[100] Park L Raman KG Lee KJ Lu Y Ferran LJ Jr. Chow WS. Suppression of accelerated diabetic atherosclerosis by the soluble receptor for advanced glycation end products. Nat Med. (1998) 4:1025–31. 10.1038/2012 9734395

[101] Wu H Zhang J Jiang X Shen X Zeng X Guo C. A soluble receptor for advanced glycation end-products attenuates myocardial ischemia/reperfusion injury via enhancing glucose metabolism. FASEB J. (2025) 39:e71173. 10.1096/fj.202502255RR 41204801

[102] Liu J Zhang J Chang J Chen L Wang H Liu Y. sRAGE inhibits myocardial ischemia/reperfusion injuries via regulating treg cells. Cell Biosci. (2025) 15:144. 41126365 10.1186/s13578-025-01482-y PMC12542090

[103] Han X Guo X Chang J Zhang J Chen L Wang H. Integrin*β*3 mediates the protective effects of soluble receptor for advanced glycation end-products during myocardial ischemia/reperfusion through AKT/STAT3 signaling pathway. Apoptosis. (2022) 27:354–67. 10.1007/s10495-022-01724-1 35359221

[104] Ramasamy R Shekhtman A Schmidt AM. RAGE/DIAPH1 and atherosclerosis through an evolving lens: viewing the cell from the “inside-out”. Atherosclerosis. (2024) 394:117304. 10.1016/j.atherosclerosis.2023.117304 39131441 PMC11309734

[105] Singh H Agrawal DK. Discovery of potential RAGE inhibitors using receptor-based pharmacophore modeling, high throughput virtual screening and docking studies. J Biotechnol Biomed. (2023) 6:501–13. 10.26502/jbb.2642-91280112 38050632 PMC10695404

[106] Dong H Chang L Tian T Shi R Yu K Wang C. Mechanism-guided pharmacotherapy for cardiometabolic multimorbidity: from pathophysiology to phenotype-prioritized treatment. Front Endocrinol. (2025) 16:1724965. 10.3389/fendo.2025.1724965 PMC12702714 41404511

[107] Prieto-Laín N Trenas A Gómez-Huelgas R Pérez-Belmonte LM. Practical approach to the management of diabetic cardiomyopathy: from molecular pathways to treatment strategies. Pol Heart J. (2025) 84:7–18. 10.33963/v.phj.110057 41496621

[108] Gao L Zhang G Kang K Xu L Zhang W Chi C. Heart failure with preserved ejection fraction (HFpEF): translational mechanisms, diagnostic evolution and therapeutic frontiers. Diabetes Res Clin Pract. (2026) 232:113116. 10.1016/j.diabres.2026.113116 41580149

[109] Farrag EAE Hammad MO Safwat SM Hamed S Hellal D. Artemisinin attenuates type 2 diabetic cardiomyopathy in rats through modulation of AGE-RAGE/HMGB-1 signaling pathway. Sci Rep. (2023) 13:11043. 10.1038/s41598-023-37678-w 37422477 PMC10329689

[110] Chen Y Meng Z Li Y. Advanced glycation end products and reactive oxygen species: uncovering the potential role of ferroptosis in diabetic complications. Mol Med. (2024) 30:141. 10.1186/s10020-024-00905-9 39251935 PMC11385660

[111] Tao A Song J Lan T Xu X Kvietys P Kao R. Cardiomyocyte-fibroblast interaction contributes to diabetic cardiomyopathy in mice: role of HMGB1/TLR4/IL-33 axis. Biochim Biophys Acta Mol Basis Dis. (2015) 1852:2075–85. 10.1016/j.bbadis.2015.07.015 26209013

